# Acoustic Immittance in children without otoacoustic emissions

**DOI:** 10.1016/S1808-8694(15)30576-0

**Published:** 2015-10-19

**Authors:** Ana Emilia Linares, Renata Mota Mamede Carvallo

**Affiliations:** 1 PhD – Experimental Physiopathology at FMUSP (University of São Paulo Medical School). Speech and Hearing Therapist - Associação dos amigos dos deficientes auditivos de Sorocaba (APADAS).; 2 Associate Professor – Speech and Therapy Program - FMUSP. Master's Degree Thesis Dissertation at the Experimental Physiopathology Program - FMUSP.

**Keywords:** electrophysiology, midlle ear, acoustic reflex, acoustic impedance tests

## Abstract

Considering the hypothesis that middle ear changes can impair the recording of otoacoustic emissions, it is possible that absent otoacoustic emissions in infants could be associated with a light tympanometric change.

**Aim:**

To study the association between transient otoacoustic emissions and changes in acoustic immittance measurements with 226Hz probe tone in neonates.

**Methods:**

Cross-sectional contemporary cohort study. 20 infants with no transient otoacoustic emissions (study group) and 101 infants with transient otoacoustic emissions (control group), with ages ranged from birth to eight months, were assessed. Infants were submitted to: admittance tympanometry; contralateral acoustic reflex threshold with stimulus of 0.5, 1, 2, 4 kHz and broad band noise; transient and distortion product otoacoustic emissions. The auditory brain response was used to study the threshold in neonates without transient otoacoustic emissions.

**Results:**

Significant statistical differences were observed between the groups (p < 0.005), characterized by reduction in tympanometric configuration and increase acoustic reflex thresholds in the study group. These data suggest the occurrence of middle ear mild impairment in infants without transient otoacoustic emissions associated with normal auditory brain response.

**Conclusion:**

tympanometry associated with acoustic reflex adds accuracy to the diagnosis of middle ear abnormalities.

## INTRODUCTION

In order to properly capture otoacoustic emissions (OAE), it is necessary to have a healthy and intact middle ear and no wax or amniotic liquid residues in the external acoustic meatus. Eustachian Tube dysfunction may also impact OEA recording.^1^, ^2^

The frequent use of OAEs as audiological investigation tool in infants has fostered even further the interest in infant tympanometry.

Both tympanometry and the investigation of acoustic reflexes make up the procedures for acoustic immitance. The contralateral acoustic reflex study helps us check the middle year all the way to the superior olivary complex.

In clinical practice, the test tone used in immittance is of 226Hz, but such procedure can be carried out using 660 and 1000Hz test tones. Studies with the 1000Hz probe in neonates proved to be efficient in detecting middle ear alterations.^3^, ^4^, ^5^

The 226Hz probe tone has been suggested in the literature as the frequency of choice to assess infants up to four months of age, because such probe would be less affected by maturity differences and also because tympanometry patterns are better interpreted in this frequency when compared to the higher frequencies.^6^, ^7^

However, in the literature, papers stress the need to be careful in carrying out this test in infants below seven months of age, because they may present a type A tympanometry curve, even when there is fluid in the middle ear.^8^

Starting from the assumption that middle ear function alterations can impair OAE recordings, it is possible that their absence in infants is associated with mild tympanometry changes.

Results from this study of impedance measures in infants can contribute to outline the procedures and to establish an identification protocol of middle ear disorders in this population, providing for the diagnosis and treatment of these alterations, before carrying out Brainstem Evoked Auditory Potential tests.

This study aimed at checking the association between Otoacoustic Emission responses and impedance alterations with the 226Hz probe in infants through a comparative analysis of both groups regarding tympanometry curve pattern and the acoustic reflex.

## METHOD

The present investigation (protocol 570/03) was submitted and approved by the Ethics Committee for project analysis.

## SERIES

The sample had infants up to eight months of age, of both genders, born at term or pre-term, with or without risk indication for hearing impairment. The infants assessed were seen in the period between April and August of 2005.

We included in the study those infants who, together with parents or guardians, received information about the research procedures, and who at the end agreed in participating and signed an informed consent form.

For the study we selected all those who did not have pinna malformations, syndromes or neurological alterations. TEOAEs present determined the inclusion of infants from the Comparison Group and the absence of TEOAEs selected the infants included in the Research Group.

Thus, 121 patients matched inclusion criteria, 101 infants in the Comparison Group and 20 in the study group.

### Equipment

- Heinne Otoscope
- AZ7 – Interacoustics Middle Ear Analyzer
- Smart - Intelligent Hearing System – Transient and Distortion Product Otoacoustic Emission Analyzer

For TEOAEs, we used a non-linear 75µsec click (oscillatory pulse). The stimulus velocity was of 19.3/s.

During the test, we presented waves 1 and 2 for correlation analysis. The amplitude from waves 1 and 2 are measured in millipascals by milliseconds. If 1 and 2 waves are overlapping and if there were strong time correlations (2 to 20 ms) and if there were strong oscillations during the time span (2 to 20ms), the TEOAE was evident. We considered waves 1 and 2 reproducibility in the following frequency bands 1k, 1.5k, 2k, 3k and 4k kHz.

For DPOAE we used two stimuli, f1 and f2 with the f2/f1 ratio of 1.22 with intensities of 65/55dBSPL respectively. The emissions were recorded in the range of 2f1-f2. We offered a maximum of 32 scans per frequency, which were presented between 500 and 8,000Hz. The test was presented in a DPGram, showing the signal to noise ratio in each frequency. Responses equal to or above 6dBSPL were deemed normal.

- SmartEP - Intelligent Hearing System (Auditory Evoked Potentials System): electronic equipment made up of a mediator computer, acoustic signal generator, amplifier and recorder. The stimulus is presented by a pair of insertion phones and bone vibrator. It has surface electrodes that capture the electrical activity coming from the structures which are part of the auditory pathway. The equipment makes an automatic calculation of wave amplitude, absolute latencies and interpeak intervals. We used click-type stimuli at 49\s presentation speed in a 20ms window. The click intensity varied between 10-99dBHL.

### Procedures

**Chart 1:**
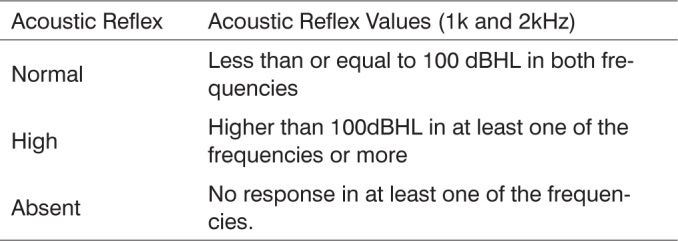
Classification of contralateral acoustic reflex for 1k and 2kHz.
- Transient Otoacoustic Emissions: with a non-linear 80dBSPL stimuli. In the ears in which response was not obtained at 80 dBSPL, a second assessment was made at 90 dBSPL, which is the intensity suggested by the equipment manufacturer. We selected the 20 milliseconds window and collected at least 100 responses and a maximum of 1024 responses. For each ear evaluated, the following Transient Otoacoustic Emission criteria were analyzed (Chart 2):

The infants were submitted to:

- Anamnesis and Informed Consent Form
- Immitance
- Tympanometry
- Acoustic reflex measure
- Transient Otoacoustic Emissions
- Distortion Product Otoacoustic Emissions
- Otorhinolaryngological clinical assessment and Brainstem Auditory Evoked Potential (infants without Transient Otoacoustic Emissions)
  Infants with auditory risk for progressive hearing loss and who had Otoacoustic Emissions remained in audiological follow up. Those who did not have auditory risk were discharged and received instructions as to hearing and language health and development.
- Acoustic reflex: To study contralateral acoustic reflex thresholds we used stimuli of 0.5k; 1k; 2k; 4 kHz; and Broad Band Noise, recorded with the 226Hz conventional probe. For the quantitative analysis of acoustic reflex results we used the values of 1k and 2kHz to classify reflex patterns, because of the possibility of having artifacts in the other frequencies, as described in Chart 1.

**Chart 2:**
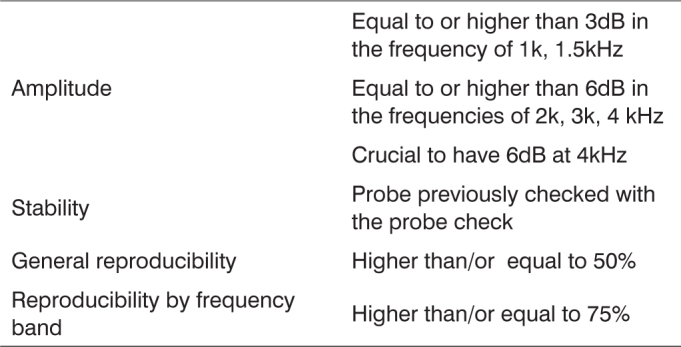
TEOAE reference values classification used in this study.

The first ear evaluated was randomly selected and the responses were collected after checking the probe fitting. It was necessary that the results attained reached the criteria aforementioned in order to consider the presence of Otoacoustic Emissions.

**Chart 3:**
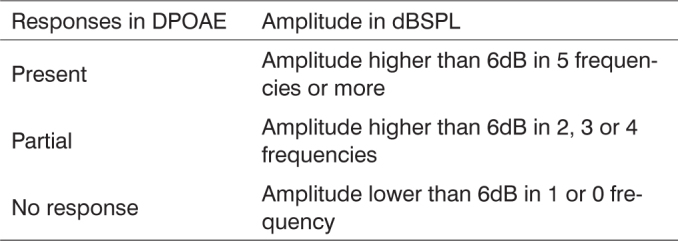
DPOAE reference values classification

In order to classify the responses from Distortion Products Otoacoustic Emissions, we used the following criteria, considering the seven frequency analysis, however disregarding the first two low frequencies (noise) (Chart 3).

The Brainstem Auditory Evoked Potential was carried out only in those infants who failed the Transient and/or Distortion Product Otoacoustic Emission test, in order to test the electrophysiological threshold. We used the click as a stimulus, initially at the intensity of 80dBHL in order to study interpeak and absolute latencies. We carried out a study with electrophysiological threshold with a stepwise reduction of 20dB to study response threshold. The latency study window was of 20ms and the stimulus velocity was of 49.1/s and 2,000 scans were recorded.^10^

Infants without Otoacoustic Emissions were submitted to otorhinolaryngological clinical evaluation.

### Statistical Method

We analyzed the data obtained from the Comparison Group infants by means of the ANOVA test, Equality of Two Ratios and Chi-Squared. In order to supplement the descriptive analysis, we used the confidence interval technique, both for the average as for the ratio.

For statistical inference analysis we defined a 0.05 significance level. And the significant values were marked with an asterisk (*).

## RESULTS

The lack of statistical difference between gender and ear, in a systematic fashion, for all electro-acoustic procedures, allowed for a comparative analysis of the response set for each group.

Thus, following we present the results from the comparative analysis between Groups, first for TOAE measures (Table 1), DPOAE (Table 2) and, afterwards, acoustic immitance measures’ analysis (Table 3).

**Table 1:** TEOAE comparison (in dBSPL) between the study and comparison groups.

TEOAE		Average	Median	Standard Deviation	Size	Lower Limit	Upper Limit	p-value
1 kHz	Study.	0.37	0.00	3.11	20	-0.69	1.44	0.002*
	Comp.	2.72	2.09	3.91	101	1.95	3.48	
1.5 kHz	Study.	1.93	0.77	4.37	20	0.44	3.42	<0.001*
	Comp.	8.35	7.78	5.58	101	7.26	9.44	
2 kHz	Study.	3.31	1.00	5.69	20	1.37	5.25	<0.001*
	Comp.	10.54	9.18	5.65	101	9.44	11.64	
3 kHz	Study.	5.68	3.69	6.71	20	3.39	7.97	<0.001*
	Comp.	13.15	12.96	5.64	101	12.05	14.25	
4 kHz	Study.	2.86	2.45	2.99	20	1.85	3.88	<0.001*
	Comp.	11.27	9.97	4.61	101	10.37	12.17	

Observation: the (*) symbol suggests a statistically significant difference

**Table 2:** DPOAEs (in dBSPL) comparison between the Study and Comparison Groups.

EOAPD		Mean	Median	Standard Deviation	Size	Lower limit	Upper limit	p-value
553Hz	Study	0.88	2.00	6.13	20	-1.21	2.97	0.786
	Comp.	1.20	1.00	5.80	101	0.07	2.33	
783Hz	Study	-0.70	0.00	3.28	20	-1.82	0.42	0.014*
	Comp.	1.79	1.00	5.44	101	0.73	2.85	
1105Hz	Study	-0.33	0.00	7.86	20	-3.01	2.35	0.011*
	Comp.	3.47	3.00	7.21	101	2.06	4.87	
1560Hz	Study	3.24	3.00	5.40	20	1.40	5.08	0.006*
	Comp.	8.02	7.00	9.35	101	6.20	9.84	
2211Hz	Study	2.76	2.00	8.36	20	-0.10	5.61	<0.001*
	Comp.	14.78	13.00	9.92	101	12.85	16.72	
3125Hz	Study	3.45	3.00	8.81	20	0.45	6.46	<0.001*
	Comp.	17.94	18.00	9.12	101	16.16	19.72	
4416Hz	Study	4.61	5.00	7.07	20	2.20	7.02	<0.001*
	Comp.	15.66	16.00	7.93	101	14.12	17.21	
6250Hz	Study	5.30	3.00	8.55	20	2.39	8.22	<0.001*
	Comp.	20.34	22.00	9.16	101	18.55	22.12	
8837Hz	Study	3.79	4.00	8.58	20	0.86	6.72	<0.001*
	Comp	16.91	17.00	9.48	101	15.06	18.76	

Observation: the (*) symbol suggests a statistically significant difference

**Table 3:** Tympanometry values between the comparison and study groups.

Ear drum	Volume (ml)		Admittance (ml)		Pressure (daPa)	
	Study.	Comp.	Study.	Comp.	Study.	Comp.
Mean	0,46	0,51	0,28	0,67	-3,64	-3,71
Median	0,40	0,50	0,30	0,70	0,00	0,00
Standard Deviation	0,12	0,21	0,27	0,27	23,02	32,48
Size	20	101	20	101	20	101
Lower limit	0,42	0,47	0,19	0,62	-11,49	-10,05
Upper limit	0,50	0,55	0,37	0,72	4,22	2,62
p-value	0,152		<0,001*		0,990	

Results from Tables 1 and 2 indicate that there are statistical differences when we compare both groups, for OAE and for the entire frequency range for TEOAE and starting at 1,105Hz for DPOAE. The Comparison Group had higher TEOAE and DPOAE amplitudes.

Table 3 shows that there is statistical difference in the immitance measures for acoustic admittance, and the Comparison Group had the higher Acoustic Admittance. As to the acoustic reflex measure (Table 4), we see that there was a difference between the groups for the entire frequency range, and the Comparison Group had the lowest acoustic reflex.

**Table 4:** Acoustic reflex threshold values (in dBHL) between the comparison and study

Reflex		Mean	Median	Standard Deviation	Size	Lower Limit	Upper Limit	p-vaue
500Hz	Study.	98.57	100.00	14.92	7	87.52	109.62	0.124
	Comp.	92.83	95.00	9.04	99	91.05	94.61	
1 kHz	Study.	102.50	102.50	9.64	8	95.82	109.18	0.004*
	Comp.	93.48	95.00	8.22	99	91.87	95.10	
2 kHz	Study.	103.33	102.50	6.83	6	97.87	108.80	0.018*
	Comp.	93.88	95.00	9.48	98	92.00	95.76	
4 kHz	Study.	111.00	115.00	10.84	5	101.50	120.50	<0.001*
	Comp.	93.21	95.00	9.99	92	91.17	95.25	
WB	Study.	104.17	102.50	7.36	6	98.28	110.06	0.027*
	Comp.	94.54	95.00	10.36	97	92.47	96.60	

Observation: the (*) symbol suggests a statistically significant difference

In comparing the types of tympanometry curves of both groups studied, we notice that for the double peak (DP) and B curve types, there is a proportionally significant difference between the groups. The presence of a type DP curve was greater in the Comparison Group (24.8%), made up of infants with TEOAE, therefore with normal hearing. However, the greater number of infants with type B tympanometry curves happened in the Study Group (33.3%). For type A, C and As tympanometry curves, we did not see differences between the groups (Table 5).

**Table 5:** Tympanogram curve type between the Study and Comparison Groups with percentage and variance values.

Curve type Cont.		Study		Comparison
A	%	48,5%		67,3%
	var	17,1%		9,1%

p-valor			0,052#	

Dp	%	3,0%		24,8%
	var	5,8%		8,4%

p-valor			0,006*	

As	%	12,1%		5,0%
	var	11,1%		4,2%

p-valor			0,153	

C	%	3,0%		1,0%
	var	5,8%		1,9%

p-valor			0,401	

B	%	33,3%		2,0%
	var	16,1%		2,7%

p-valor			<0,001*	

The 20 children in the study group, without Transient Otoacoustic Emissions, were submitted to a Brain Stem Auditory Evoked Potential and otorhinolaryngological evaluation. Results revealed that 75% of them had conductive hearing alteration, 15% had high frequency hearing loss, 5% had mild/moderate sensorineural hearing loss, and 5% had moderate and profound sensorineural hearing loss.

## DISCUSSION

The major goal of the present investigation was to study the relationship between the OAEs and the immitance findings in infants in an attempt to acquire information that help in the diagnostic decision, based on the auditory evaluation by OAEs and immitance findings. Very little was discovered about immitance responses at this age range. Screening for middle ear function in children is still not broadly studied.^11^

The greatest difficulty found by researchers is to standardize tympanometry in infants, because when in face of no OAEs, during neonatal screening or during a diagnosis process, the concern is to differentiate between middle and inner ear impairment.^12^, ^13^

The significant difference between the groups (p<0.005) for TEOAE was expected, having in mind the inclusion criteria for the groups, TEOAE present or absent. We then decided to include TEOAE between the groups in order to analyze it by frequency and illustrate the difference between the groups. For TEOAE, this significant difference remained, showing matching results between the two types of OAEs.^14^, ^15^

The comparison group showed higher TEOAE and DPOAEs in relation to the study group.

Pressure changes in the middle ear can interfere in the amplitude response of both TEOAE and DPOAEs.^2^ In the present investigation, we observed a difference between the two groups in relation to the tympanometry curve height, and the group with no TEOAEs had the lowest admittance peak value: 0.28ml, while the comparison group had a value of 0.67ml, as depicted on Table 3.

These results suggest a mild alteration in the middle ear of the study group, considering the tympanometry curve's low value. Admittance reduction is associated with a lower tympano-ossicles mobility, characterizing an alteration in the sound mechanical conduction through the middle ear.

The middle ear alteration causes a lot of loss of the sound presented to the external acoustic meatus, which follows towards the cochlear, as well as cochlear OAEs response attenuation in the external acoustic meatus.^16^

Middle ear effusion can occur in 50% of the neonate ears who fail OAEs hearing screening. The authors consider middle ear effusion as a severe and significant cause of OAEs hearing screening failure in newborns at the intensive care units.^17^ The present investigation carried out with infants found a conductive alteration, confirmed by the otorhinolaryngological clinical evaluation and by the Brain Stem Auditory Evoked Potential in 75% of those who failed TEOAEs (study group).

In the group with TEOAEs, most infants presented a type A tympanometry curve, and a considerable number of them (24.8%) presented a pattern commonly found in neonates and infants – the Double Peak Pattern.

The Double Peak tympanometry pattern was considered a normal response pattern for the population of newborns, even with the 226Hz probe. 7, 18, 19

The study carried out with 50 infants with age between birth and eight months also identified Double Peak Tympanograms in 10.31% of the ears studied, using the 226Hz probe.^7^

Other studies also proved a Double Peak tympanogram, explaining such occurrence based on the fact that the neonate auditory system is ruled by the mass effect.^20^

If we consider a purely neonatal age range, the rate of Double peak tympanograms increases considerably (52.3%).^5^

In the group of infants without TEOAEs, there was a higher rate of tympanogram alterations as presented on Table 5. We noticed that in 48.5% of altered and normal tympanograms, only 3% had a Double peak curve.

Thus, comparing tympanogram findings between the groups shows that there was a statistically difference as to the tympanogram curve distribution. We observed a higher occurrence (24.8%) of Double peak tympanometry patterns in the TEOAEs group when compared to the study group. In relation to the B-type tympanogram, it was more common in the group without TEOAEs. Considering that the double peak and the type A patterns suggest normal middle ear function, the comparison group gathered 88% normal tympanogram results, while the study group presented only 51.5% normal tympanograms.

The Double Peak Tympanogram happens in the middle ear resonance frequence.^16^ Newborns and infants present, in the middle ear, the resonance frequency shifted to the lower frequencies.^21^ Middle ear alterations, in the study group, may have altered this resonance frequency pattern, generating only 3% of double peak tympanograms.

One of the major difficulties is to analyze the tympanogram curve profile, especially with high frequency probes (678 and 1000Hz). Findings from the present investigation, carried out with 121 infants, showed that 100% of the tympanogram curves could be classified.^22^

The usefulness of tympanometry has been clearly established in the entire population, except in children below six months of age. The authors reported that studies carried out on infant tympanograms, with high frequency probes, described the high number of non-analyzable tympanometry curves, such as asymmetric and inverted tympanometry curves. The 226Hz probe suffers less influence from the middle ear maturity aspects. The results from the present investigation corroborate this statement.^6^

The results shown on Table 4 indicate the presence of an acoustic reflex in 96.5% of the comparison infants in the present investigation. Another paper showed that 100% of normal hearing infants presented an acoustic reflex, suggesting this test as an indicator of hearing pathways integrity when associated to normal behavioral hearing evaluation.^7^, ^16^

The acoustic reflex value in the neonatal and infant populations is a feasible and doable evaluation method, which can contribute with information on the auditory pathway integrity.^7^, ^23^ However, few papers have been published about this method in the evaluation of this population.

Among the children without TEOAEs, there was a middle ear alteration in 75% of the ears, and no acoustic reflex in 100% of them. Thus, the acoustic reflex contributes, together with the otorhinolaryngological and electrophysiological evaluation, in order to determine, hearing alterations.

These results match the ones published^17^, which state that middle ear effusion could happen in 50% of neonate ears who fail hearing screening with OAEs.

Correlating them with immitance results from both groups, we notice that the type of curve was not the only sign of middle ear alteration. The reduced tympanometry height (admittance intensity) proved to be an indicator of middle ear alteration in this population, together with the lack of an acoustic reflex.

These results help us associate TEOAEs and acoustic admittance reduction, shown by the 226Hz tympanometry and the increase in the acoustic reflex threshold.

## CONCLUSION

Data from the present investigation allows us to draw the following conclusions:

• Curve type is not the only sign of middle ear alteration. Lower tympanometry curve height (admittance intensity) seems to be an indication of middle ear alteration in this population. Children without TEOAE had lower tympanometry curve height.
• The combined use of tympanometry and acoustic reflex in infants adds precision to the diagnoses of middle ear alteration.
• There was a predominance of middle ear alteration in the group without TEOAEs.
